# IGF2BP3 From Physiology to Cancer: Novel Discoveries, Unsolved Issues, and Future Perspectives

**DOI:** 10.3389/fcell.2019.00363

**Published:** 2020-01-15

**Authors:** Caterina Mancarella, Katia Scotlandi

**Affiliations:** Laboratory of Experimental Oncology, IRCCS Istituto Ortopedico Rizzoli, Bologna, Italy

**Keywords:** RNA-binding protein, IGF2BP3, embryonic development, cancer, biomarker

## Abstract

RNA network control is a key aspect of proper cellular homeostasis. In this context, RNA-binding proteins (RBPs) play a major role as regulators of the RNA life cycle due to their capability to bind to RNA sequences and precisely direct nuclear export, translation/degradation rates, and the intracellular localization of their target transcripts. Alterations in RBP expression or functions result in aberrant RNA translation and may drive the emergence and progression of several pathological conditions, including cancer. Among the RBPs, insulin-like growth factor 2 mRNA-binding protein 3 (IGF2BP3) is of particular interest in tumorigenesis and tumor progression. This review highlights the molecular mechanisms underlying the oncogenic functions of IGF2BP3, summarizes the therapeutic potential related to its inhibition and notes the fundamental issues that remain unanswered. To fully exploit IGF2BP3 for tumor diagnosis and therapy, it is crucial to dissect the mechanisms governing IGF2BP3 re-expression and to elucidate the complex interactions between IGF2BP3 and its target mRNAs as normal cells become tumor cells.

## Introduction

RNA-binding proteins (RBPs), along with microRNAs (miRNAs; Box 1) and long non-coding RNAs (lncRNAs; Box 1), dictate the entire RNA life cycle from alternative splicing to nuclear export, transcript storage, stabilization, subcellular localization and degradation (for a review, please consider Coppin et al., 2018), thus representing major cotranscriptional and/or posttranscriptional regulators of gene expression. In humans, 1393 RBPs, which account for 7.5% of the proteome, have been recently identified (Hentze et al., 2018). Each contemporary RBP binds to hundreds of RNAs, including both coding and non-coding RNAs, and affects their expression and translation, thus playing a wide regulatory role in practically all physiological processes. Accordingly, the deregulation of RBPs frequently occurs in pathological conditions, particularly cancer (Coppin et al., 2018). Recent next-generation sequencing analyses in tumor specimens have demonstrated that genes encoding RBPs have significantly higher overexpression than non-RBP-coding genes (Neelamraju et al., 2018). In addition, evidence has consistently shown that RBPs are strongly implicated in the regulation of most cancer hallmarks, such as cell proliferation, resistance to cell death, stemness, cell dissemination, and immune system evasion, and may act as promising biomarkers of tumor progression (Pereira et al., 2017).

At least 16 families of RBPs are deregulated in cancer (Pereira et al., 2017). Of those, the highly conserved family of insulin-like growth factor 2 mRNA-binding proteins (IGF2BPs), which includes the paralogs IGF2BP1, IGF2BP2, and IGF2BP3, primarily play oncogenic roles in cancer. Over the past few years, studies have increasingly documented the contribution of IGF2BPs to fundamental processes in cancer biology, and their overexpression has been widely associated with adverse patient outcomes in many different tumors. This family was named IGF2BPs because, originally, the three members were identified as posttranscriptional regulators of the fetal growth factor IGF2 (Nielsen et al., 1999). Structurally, IGF2BP1, IGF2BP2, and IGF2BP3 share a 59% amino acid sequence identity, which reaches 73% between IGF2BP1 and IGF2BP3. These family members are characterized by a peculiar structure composed of the following six RNA-binding domains: two RNA recognition motifs (RRMs; Box 1) in the N-terminal region and four K-homology (KH; Box 1) domains in the C-terminal region arranged in three pairs of didomains (RRM1 + 2, KH1 + 2, and KH3 + 4) and separated by flexible linkers (Jia et al., 2018). Overall, all four KH domains contribute to RNA-binding, ribonucleoprotein (RNP) granules formation (Box 1), and cellular localization (Wachter et al., 2013). In general, IGF2BPs bind to their target RNAs at the 5′-UTR, 3′-UTR or coding regions (Box 1) by recognizing specific RNA motifs, such as the first identified CAUH (H = A, U, C) (Hafner et al., 2010). In addition, posttranscriptional modifications of target RNAs, such as the *N*^6^-methyladenosine modification (Box 1), render modified RNAs more attractive for IGF2BP binding (Huang et al., 2018b). Approximately 55–70% of the recognized target RNAs are shared among the three proteins (Huang et al., 2018b). Accordingly, IGF2BPs can form homodimers and heterodimers on target RNAs, partially explaining the observed overlap among the recognized targets (Nielsen et al., 2004; Hammerle et al., 2013). Physiologically, the IGF2BPs are expressed during embryogenesis but are absent in adult tissues, except for IGF2BP2, which is mainly involved in metabolic processes and is maintained in most normal tissues (Nielsen et al., 1999; Dai et al., 2015). IGF2BPs are mainly localized in the cytoplasm (Nielsen et al., 1999), but some evidence also demonstrates their presence in the nucleus, where they directly bind to target RNAs after transcription and shuttle them between the nucleus and cytoplasm. The nuclear role of IGF2BPs is further demonstrated by the identification of nuclear export signals within the RNA-binding KH2 and KH4 domains (Nielsen et al., 2003; Oleynikov and Singer, 2003; Rivera Vargas et al., 2014). However, the exact mechanisms governing the nuclear localization of IGF2BPs still need elucidation. For a more general introduction to the phylogenetic origin, gene/mRNA/protein structure and expression pattern of the IGF2BPs in normal or pathological tissues, readers are referred to several excellent reviews (Bell et al., 2013; Lederer et al., 2014; Degrauwe et al., 2016b; Cao et al., 2018).

**BOX 1. Glossary.**

**Chromatin immunoprecipitation (ChIP)**: method for the identification of transcription factor DNA target, based on DNA/proteins crosslinking, immunoprecipitation of the transcription factor of interest, DNA extraction, and qRT-PCR or sequencing

**circularRNAs (circRNAs)**: large class of non-coding RNAs deriving from a non-canonical alternative splicing called “backsplicing” and characterized by a covalent link between the 3′ and 5′ ends

**K-homology (KH) domains**: RNA-binding domain of ∼70 amino acids which forms a three-stranded β-sheet packed against three α-helices, which recognizes both DNA and RNA throught a conserved GXXG loop

**Locasomes**: large, motile RNP granules containing untranslated mRNAs and acting as cytoplasmic repository for transcripts

**Long non-coding RNAs (lncRNAs)**: non-coding transcripts larger than 200 nucleotides regulating gene expression

**microRNAs (miRNAs)**: class of short non-coding RNAs (19-25 nucletides) regulating posttranscriptional silencing of target transcripts

**N^6^-methyladenosine modification**: epigenetic RNA modification influencing mRNA fate including stability, splicing, and translation rate

**Photoactivatable ribonucleoside-enhanced crosslinking and immunoprecipitation (PAR-CLIP)**: method for mapping RBP/RNA interaction, based on incorporation of photoreactive nucleosides in newly transcribed RNAs followed by UV crosslinking between transcripts and RBPs, immunoprecipitation of the RBP of interest and RNA extraction. Upon reverse transcription, sequencing analysis is performed

**Processing bodies (P-bodies)**: cytoplasmic RNP granules composed by mRNAs and proteins with a role in translation repression and mRNA decay

**Ribonucleoprotein (RNP) granules**: cytoplasmic protein/RNA assemblies acting as posttranscriptional regulators of gene expression

**RNA immunoprecipitation (RIP)**: method for RBP RNA target identification, based on immunoprecipitation of the RBP of interest followed by RNA extraction, reverse transcription and qRT-PCR or sequencing analysis

**RNA-induced silencing complex (RISC)**: cytoplasmic complex incorporating miRNAs for the recognition and degradation of complementary mRNAs

**RNA-recognition motifs**: RNA-binding domain of ∼ 80–90 amino acids which folds in two α-helices packed against a four-stranded anti-parallel β-sheet, involved in RNA recognition

**Stress granules**: cytoplasmic RNP granules, composed by mRNA, proteins and 40S ribosome subunits, that are induced under stress conditions and where transcripts are stabilized and translation is silenced

**Untranslated region (UTR)**: sequences of mature mRNA, located upstream (5′-UTR) or downstream (3′-UTR) from the coding region, holding post-transcriptional regulatory elements that affect gene expression

This review focuses on IGF2BP3 and its role in human cancer, highlighting the contradictions and discrepancies related to its still poorly understood mechanisms of action and the potential of this protein as diagnostic, prognostic and therapeutic biomarker.

## IGF2BP3

**FIGURE 1:**
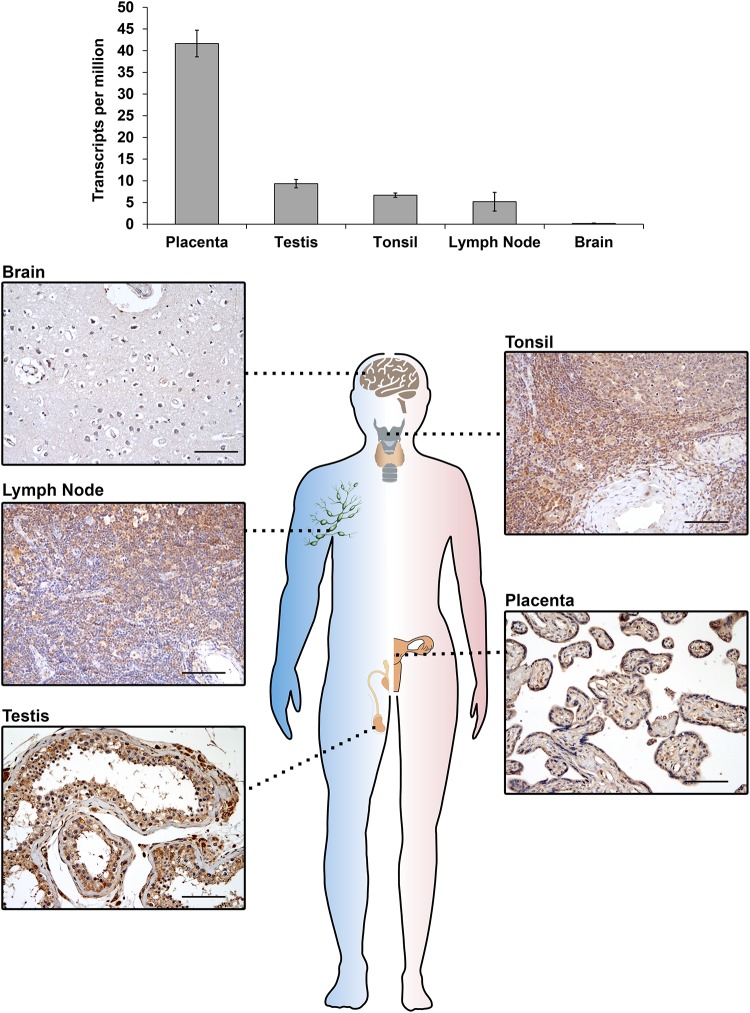
IGF2BP3 mRNA **(top)** and protein **(bottom)** expression detected by RNA-seq or immunohistochemical analyses in normal human tissue samples. RNA-seq data are courtesy of the Human Protein Atlas, www.proteinatlas.org (Uhlen et al., 2015). For immunohistochemistry, an anti-IGF2BP3 primary antibody (Santa Cruz, cat.# sc-47893; dilution 1/50) was utilized. A scale bar of 100 μm is shown.

The *IGF2BP3* gene (also known as *IMP3*, *KOC*, *CT98*, *KOC1*, and *VICKZ3*) is located on chromosome 7p15.3 in humans (Monk et al., 2002) and encodes a 69 kDa protein; this gene was first identified by Mueller-Pillasch et al. (1997) to be overexpressed in pancreatic cancer. IGF2BP3 expression was subsequently observed in mouse embryos (Mueller-Pillasch et al., 1999; Nielsen et al., 2002), but the physiological effects elicited by IGF2BP3 in these tissues are still elusive mostly due to the lack of available knockout *in vivo* models. Insight regarding the putative IGF2BP3 peculiar functions in normal embryonic development is based on studies investigating its ortholog Vg1-RBP in *Xenopus laevis*, which shares an 84% amino acid identity with human IGF2BP3. The loss of Vg1-RBP causes an abnormal head morphology, the lack of a lens and dorsal fin, a curved neural tube, the absence of the roof plate in the neural tube (Yaniv et al., 2003), impaired gut morphogenesis, the loss of pancreatic organogenesis (Spagnoli and Brivanlou, 2006) and the lack of meiotic maturation (Git et al., 2009). These data, which insinuate that IGF2BP3 plays a general role in neural development and organogenesis, are overall consistent with evidence concerning the spatial distribution of IGF2BP3 expression during advanced stages of gestation (E11.5–E12.5) in embryonic mice, indicating that this RBP is present in neural cells, the intestine, thymus, pancreas, and kidney epithelial germ layers (Mueller-Pillasch et al., 1999; Mori et al., 2001; Hansen et al., 2004). Evidence in humans is even more limited but confirms the major role of IGF2BP3 as an embryonic regulator. Accordingly, fetal hematopoietic progenitors, including megakaryocytes, express IGF2BP3 at higher levels than their adult counterparts, and IGF2BP3 contributes to the maintenance of the molecular and phenotypic features of fetal-type cells (Elagib et al., 2017). Accumulating data indicate that IGF2BP3 is also present in mature tissues (Hammer et al., 2005; Burdelski et al., 2018). In adult mice, IGF2BP3 is measurable in the lungs, spleen, muscles, gut, pancreas, kidneys, brain, ovaries and testes (Hammer et al., 2005; Bell et al., 2013). In human adult tissues, IGF2BP3 is detectable in the placenta, lymph nodes, tonsils, and testes (Figure 1), confirming an intriguing but still unclear association between IGF2BP3 and reproductive organs. Sporadic evidence regarding the role of IGF2BP3 role in human adult tissues shows that IGF2BP3 drives normal placental development through the correct migration of trophoblast cells into the maternal decidua in both *in vivo* and *ex vivo* models (Haouzi et al., 2011; Li et al., 2014).

Sexual dimorphism has been barely investigated for this RBP. IGF2BP3 mRNA expression in the mouse gonads appeared higher in testes than in ovaries (Hammer et al., 2005). A direct comparison between IGF2BP3 expression and sex was performed in the brains of zebrafish, but no differential expression was found in male versus female individuals (Arslan-Ergul and Adams, 2014).

**FIGURE 2:**
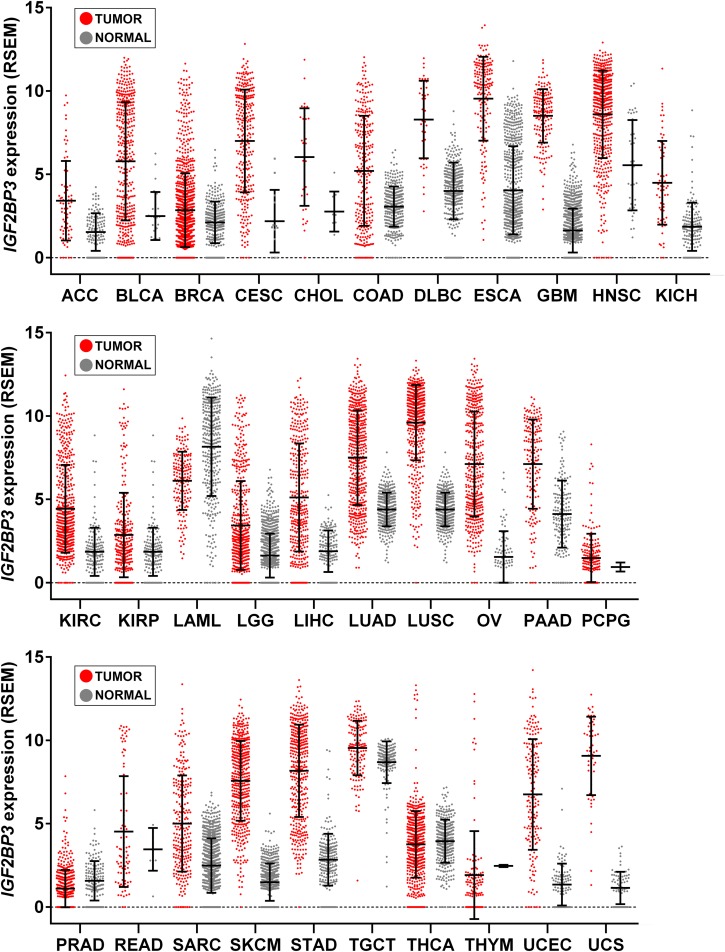
*IGF2BP3* gene expression across human tissue and cancer types. Scatter plots showing *IGF2BP3* levels from The Cancer Genome Atlas (TCGA), Genotype-Tissue Expression (GTEx), and Target projects obtained from the UCSC Xena browser (Goldman et al., 2019). Data are RSEM normalized. Mean ± standard deviation is shown. LAML, Acute Myeloid Leukemia; ACC, Adrenocortical carcinoma; BLCA, Bladder Urothelial Carcinoma; LGG, Brain Lower Grade Glioma; BRCA, Breast invasive carcinoma; CESC, Cervical squamous cell carcinoma and endocervical adenocarcinoma; CHOL, Cholangiocarcinoma; COAD, Colon adenocarcinoma; ESCA, Esophageal carcinoma; GBM, Glioblastoma multiforme; HNSC, Head and Neck squamous cell carcinoma; KICH, Kidney Chromophobe; KIRC, Kidney renal clear cell carcinoma; KIRP, Kidney renal papillary cell carcinoma; LIHC, Liver hepatocellular carcinoma; LUAD, Lung adenocarcinoma; LUSC, Lung squamous cell carcinoma; DLBC, Lymphoid Neoplasm Diffuse Large B-cell Lymphoma; OV, Ovarian serous cystadenocarcinoma; PAAD, Pancreatic adenocarcinoma; PCPG, Pheochromocytoma and Paraganglioma; PRAD, Prostate adenocarcinoma; READ, Rectum adenocarcinoma; SARC, Sarcoma; SKCM, Skin Cutaneous Melanoma; STAD, Stomach adenocarcinoma; TGCT, Testicular Germ Cell Tumors; THYM, Thymoma; THCA, Thyroid carcinoma; UCS, Uterine Carcinosarcoma; UCEC, Uterine Corpus Endometrial Carcinoma.

Transgenic overexpression of IGF2BP3 was performed in mice to shed light on the effects of re-expression of this protein in adult tissues. Interestingly, transgenic mice displayed extensive remodeling of the exocrine pancreas, leading the pancreas to resemble embryonic tissues, with increased acinar cell proliferation, a reduction in the acinar cell compartment, and the appearance of interstitial cells with a dual differentiation capacity (Wagner et al., 2003). Overall, these features corresponded to acinar-to-ductal metaplasia, which represents a major origin of the pancreatic preneoplastic lesions that eventually develop into pancreatic ductal adenocarcinoma, in both humans and in mice (Chuvin et al., 2017). More recently, Palanichamy et al. (2016) created an *in vivo* model of IGF2BP3-enforced expression in a murine hematopoietic system and observed increased hematopoietic stem and progenitor cell proliferation, skewed hematopoietic development to the B cell/myeloid lineage, atypical B cell infiltration into the thymic medulla, and increased myeloid cells in the spleen, features similar to those seen early in leukemogenesis. Beyond indicating the capability of IGF2BP3 to recapitulate a fetal-like phenotype, these evidences suggest a putative role of IGF2BP3 in tumorigenesis since the *de novo* expression of RBP in adult tissues apparently provides a favorable context for the emergence of neoplastic lesions. Accordingly, IGF2BP3 is detectable in some premalignant human lesions, including dysplasia in Barrett esophagus (Gadara et al., 2017), pancreatic intraductal neoplasia (Wang et al., 2015), and atypical endometriosis (Vercellini et al., 2013); in addition, many tumor types upregulate IGF2BP3 compared to normal tissue counterparts (Figure 2).

## Regulation of IGF2BP3 Expression in Cancer

**FIGURE 3:**
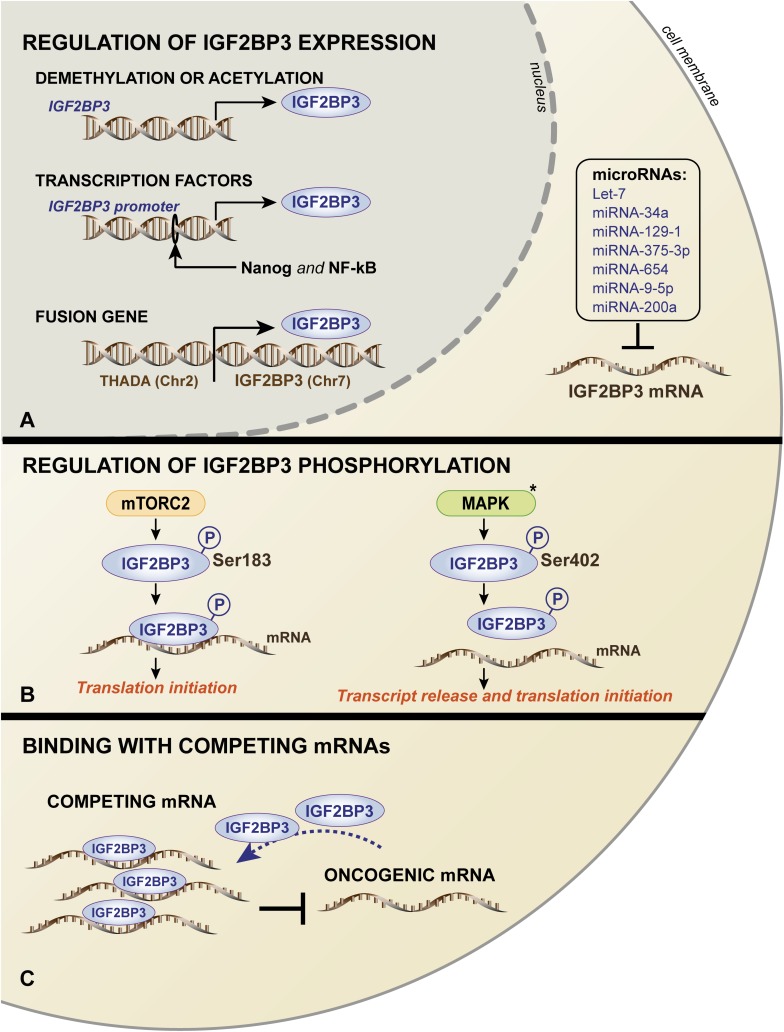
Schematic representation of the mechanisms governing IGF2BP3 expression **(A)** and functions **(B,C)**. **(A)** In the nucleus, IGF2BP3 transcription is regulated by (i) DNA methylation or the acetylation of the *IGF2BP3* gene; (ii) activation of the *IGF2BP3* promoter by transcription factors, such as Nanog and NF-kB; and (iii) occurrence of chromosomal translocation. In the cytoplasm, *IGF2BP3* mRNA is regulated by several microRNAs. IGF2BP3 functions are controlled by **(B)** mTORC2- or MAPK-mediated phosphorylation, which influences the translation of its target mRNAs, and **(C)** competing, non-oncogenic mRNAs that may prevent the interaction between IGF2BP3 and its oncogenic transcript targets. The black asterisk (^∗^) indicates that the process was described in the ortholog Vg1-RBP in *Xenopus Laevis*.

Very limited information regarding the molecular regulatory mechanisms responsible for human IGF2BP3 expression is available. The mechanisms include genomic alterations, epigenetic and transcriptional control, and post-translational modifications/interactions, summarized in a schematic in Figure 3.

Mutations in RBP coding genes are rare. Germline mutations affecting the coding regions of RBPs occur in less than 1% of all proteins, while only 15% of RBPs across solid tumors are mutated in the protein sequence (Sebestyen et al., 2016; Pereira et al., 2017). Accordingly, to date, mutations in the *IGF2BP3* gene have not been described, and gene amplification has been observed in less than 20% of lung adenocarcinoma, pancreatic, and bladder cancers (Panebianco et al., 2017). Furthermore, 5% of thyroid tumors and 25% of pancreatic cancers hold a specific balanced chromosomal translocation between the IGF2BP3 chromosomal locus on 7p15.3 and the actively transcribed THADA locus on 2p21, which results in the strong overexpression of IGF2BP3 (Panebianco et al., 2017).

Other mechanisms include DNA methylation and acetylation processes. Demethylated CpG islands characterize the *IGF2BP3* promoter in intrahepatic cholangiocarcinoma cases, which were in stark contrast to normal liver tissues that were heavily methylated (Gao et al., 2014). More recently, a large-scale sequencing analysis of datasets of 15 cancer types from The Cancer Genome Atlas (TCGA) confirmed these data, showing an inverse correlation between the DNA methylation status of the *IGF2BP3* promoter and *IGF2BP3* mRNA expression (Panebianco et al., 2017). Consistently, the treatment of murine osteosarcoma cells with a DNA methyltransferase inhibitor or histone deacetylase inhibitors resulted in a significant upregulation of IGF2BP3 expression (Ueki et al., 2012).

In addition, the increased transcriptional activation of the *IGF2BP3* promoter has been attributed to the binding of aberrantly expressed transcription factors. Chromatin immunoprecipitation (ChIP) assays (Box 1) confirmed the direct binding of the transcription factors Nanog and NF-κB to the *IGF2BP3* promoter, thus sustaining its expression in tumor cells and favoring the stemness and migration properties, respectively (Chen et al., 2013; Bhargava et al., 2017). In triple-negative breast cancer cells, EGFR signaling regulates *IGF2BP3* transcription since the *IGF2BP3* promoter activity decreased after MEK1/2 signaling inhibition downstream of EGFR (Samanta et al., 2012).

At posttranscriptional level, several miRNAs regulate IGF2BP3 in different tumor types. In particular, *IGF2BP3* expression is inhibited by the let-7 family of miRNAs (Mayr et al., 2007; Fawzy et al., 2016; Kugel et al., 2016; JnBaptiste et al., 2017; Lin et al., 2017), miRNA-34a (Zhou et al., 2017), miRNA-129-1 (Kouhkan et al., 2016), miRNA-375-3p (Cen et al., 2018), miRNA-654 (Jin et al., 2018), miRNA-9-5p (Canella et al., 2015), and miRNA-200a (Kim et al., 2018).

In addition to regulating the expression of IGF2BP3, intracellular signaling mechanisms may impact its function. IGF2BP3 activity can be influenced by mTOR, which is a major downstream effector of the phosphoinositide 3-kinase (PI3K) (Fruman et al., 2017) and/or mitogen-activated protein kinase (MAPK) pathway (Liu et al., 2018). In humans, the Ser183 residue, which is located between the RRM2 and KH1 domains of IGF2BP3, has been indicated as a phosphorylation site of mTORC2. It has been suggested that IGF2BP3 undergoes phosphorylation during translation and, importantly, that the phosphorylated status enhances IGF2BP3 binding to the 3′-UTR of *IGF2*, leading to translation initiation of *IGF2* mRNA and increased IGF2 expression (Dai et al., 2013). Therefore, a positive feedback loop may exist between the IGF/PI3K/MAPK/mTOR pathway and IGF2BP3 expression in cancer cells. In *X. laevis*, similar studies were conducted to investigate the ortholog Vg1-RBP. These studies demonstrated that Vg1-RBP is phosphorylated by the MAPK mediator Erk2 at residue S402, which is located in the linker between the KH1 + 2 and KH3 + 4 didomains and represents a crucial modification for the release of its mRNA target Vg1 during meiotic maturation (Mueller-Pillasch et al., 1999; Git et al., 2009).

Finally, an interaction with endogenous competing RNAs, including mRNAs or lncRNAs, was reported as an alternative mechanism regulating RBPs activity (Kim et al., 2016). Consistently, as observed in Ewing sarcoma cells, the functions of IGF2BP3 can be limited by the mRNA expression of *ABCF1*, which is a partner transcript of IGF2BP3. *ABCF1* mRNA can associate with IGF2BP3 and limit its interaction with oncogenic target transcripts, thus acting as a sponge to repress the oncogenic function of IGF2BP3 (Mancarella et al., 2018b).

## Molecular Mechanisms of Action of IGF2BP3

### Specificities of IGF2BP3 With Respect to the Other IGF2BPs

IGF2BPs share many common features; however, the three proteins are not functionally redundant because they do not recognize RNAs with the same affinity or recognize the same RNAs. Accordingly, 30–50% of the target RNAs are specifically regulated by each family member (Chao et al., 2010; Huang et al., 2018b). These specificities mainly rely on the different RNA-binding properties displayed by each IGF2BP. For instance, IGF2BPs bind to sites within the 3′-UTR more frequently than they bind to sites within the 5′-UTR (Huang et al., 2018b). Nevertheless, IGF2BP3 binds to coding regions with a higher frequency than either IGF2BP1 or IGF2BP2 (Conway et al., 2016). In addition, a recent analysis of RNA recognition by multidomain IGF2BP proteins indicated that while IGF2BP1 associates with the CGGAC RNA motif, IGF2BP3 recognizes two related GGC-core elements (GGCA and CGGC), further supporting the existence of differences during the recognition of RNA (Schneider et al., 2019). The data described in the literature suggest that multiple RNA motifs, including CACA, UACA, AACA (Conway et al., 2016), GCAC (Palanichamy et al., 2016), and GGAC (Huang et al., 2018b), are recognized by the IGF2BP family, but the extent to which these sequences are specific to each RBP is still unclear. These differences are possibly due to distinct paralog-specific biochemical properties of the RNA-binding domains. While all four KH domains were identified as relevant for RNA binding, recent evidence demonstrates a crucial contribution of both the RRMs (Jia et al., 2018) and the KH domains (Wachter et al., 2013) to IGF2BP3, adding an additional element of diversity separating this RBP from its paralogs.

Another major difference among the paralogs relies on their mechanism of action on target RNAs. In the cytoplasm, IGF2BP1 and IGF2BP3 form large (200–700 nm optical diameter), motile ribonucleoprotein (RNP) granules named locasomes (Box 1), which are located beneath the plasma membrane in the perinuclear region or the lamellipodia of the leading edge depending on the cell type and cell confluence (Nielsen et al., 2002; Weidensdorfer et al., 2009). These granules represent a unique entity, that is distinct from processing bodies (P-bodies) and stress granules (Box 1; Jonson et al., 2007; El-Naggar and Sorensen, 2018; Luo et al., 2018). Locasomes lack 60S ribosomal units; elongation factors, such as eIF4E and eIF4G (Jonson et al., 2007; Weidensdorfer et al., 2009); and the RNA-induced silencing complex (RISC; Box 1; Jonson et al., 2014), indicating that these granules serve as a protected cytoplasmic repository for IGF2BP target transcripts. However, differences still exist between IGF2BP1 and IGF2BP3 because IGF2BP3 was also observed to recruit RISC to locasomes (Ennajdaoui et al., 2016), further adding another level of complexity and heterogeneity to the action of these closely related molecules. The mechanisms of action of IGF2BP2 are less defined but may putatively differ since this protein was found to interact with P-bodies (Degrauwe et al., 2016a, b), which are cytoplasmic RNP granules mainly involved in mRNA decay.

### Mechanistic Events

**FIGURE 4:**
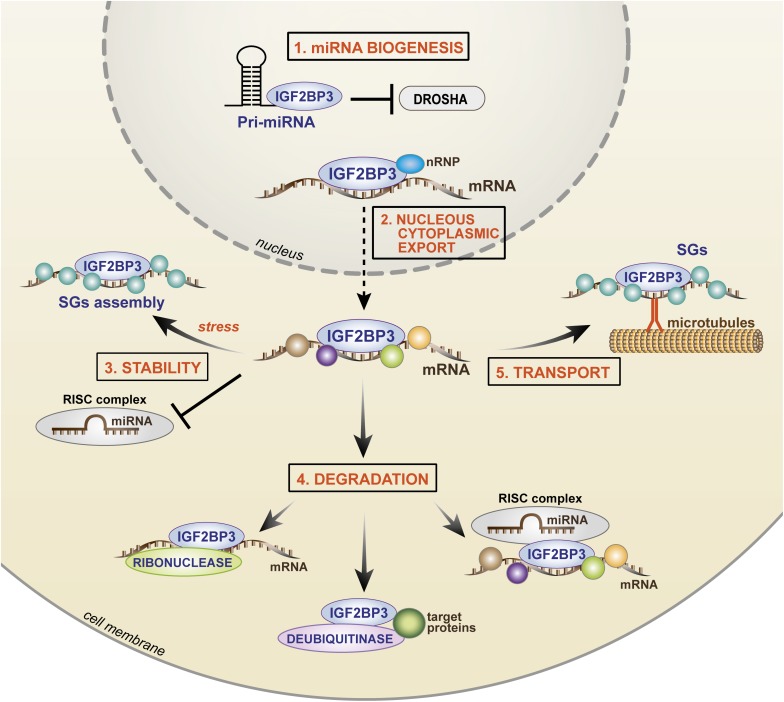
Cartoon depicting the mechanisms of IGF2BP3 activity. In the nucleus, **(1)** IGF2BP3 drives microRNA biogenesis, preventing the binding of Drosha to the pri-miRNA and **(2)** IGF2BP3 interacts with nRNP and it binds target transcripts favoring nuclear export. In the cytoplasm, IGF2BP3 acts within ribonucleoprotein granules as depicted, thus controlling **(3)** stability, **(4)** degradation, or **(5)** transport of its target mRNAs. For stability, IGF2BP3 can prevent the activity of RISC or recruit SG proteins under stress conditions. For degradation, IGF2BP3 can recruit RISC or directly interact with enzymes, such as ribonuclease or deubiquitinase, thus inducing microRNA-dependent and microRNA-independent target degradation. For transport, IGF2BP3 is assembled in SGs with its target mRNAs and drives their localization along microtubules toward areas of active translation. nRNP, nuclear ribonucleoprotein; SGs, stress granules; RISC, RNA-induced silencing complex.

RNA immunoprecipitation and sequencing (RIP-seq; Box 1) and photoactivatable ribonucleoside-enhanced crosslinking and immunoprecipitation (PAR-CLIP; Box 1) approaches indicate that ∼1000 to ∼4000 transcripts are bound by IGF2BP3 in humans (Jonson et al., 2014; Ennajdaoui et al., 2016; Palanichamy et al., 2016; Huang et al., 2018b). Among these, IGF2BP3 regulates RNA stability, RNA degradation, RNA localization (Bell et al., 2013), and miRNA biogenesis, but the exact molecular processes governing these functions have only begun to be elucidated. The current knowledge regarding IGF2BP3 action in cancer cells is summarized in Figure 4.

A major mechanism of IGF2BP3 activity is based on its complex interaction with the miRNA machinery (Degrauwe et al., 2016b; Trabucchi and Mategot, 2019). Both RBPs and miRNAs converge on the 3′-UTR of mRNAs, and the juxtaposition of their binding contributes to the combinatorial mechanisms of posttranscriptional gene regulation with a relevant impact on cellular fate and behavior. IGF2BP3 promotes mRNA stability/degradation by interacting with miRNAs through different processes as follows: (1) IGF2BP3 may protect target mRNAs from miRNA-dependent degradation by segregating transcripts into cytoplasmic RNP granules that do not contain RISC (Jonson et al., 2014); (2) IGF2BP3 may modulate the association between target transcripts and RISC (Ennajdaoui et al., 2016); (3) IGF2BP3 may compete with miRNAs for common binding sites on the 3′-UTRs of target transcripts (Ennajdaoui et al., 2016); and (4) IGF2BP3 may affect miRNA biogenesis, thus indirectly affecting the fate of miRNA targets (Wang et al., 2019).

The best described example of IGF2BP3 activity is its opposing effect on let-7 miRNA action. IGF2BP3 has been shown to segregate *HMGA2* and *LIN28B* transcripts and other let-7 targets into RISC-free RNP granules (locasomes), thereby protecting them from let-7-dependent silencing and providing generalized protection from miRNAs, including the activity of miR-181a/b (Jonson et al., 2014; Degrauwe et al., 2016b). Therefore, RISC-free locasomes represent a cytoplasmic shelter (“safe house”) in which oncogenes are protected from degradation. However, much still needs to be learned: the mechanism by which RISC is excluded from these granules; the specificity of the IGF2BP3 locasome composition; mechanisms underlying the interaction between IGF2BP3 and RISC. A study conducted by Ennajdaoui et al. (2016) revealed the bimodal capability of IGF2BP3 to regulate mRNA fate because, on one side, the RBP is able to compete with miRNAs for common binding sites on target transcripts to avoid binding to the RISC complex; on the other side, IGF2BP3 can promote the association between mRNAs and RISC, thus favoring mRNA degradation.

Overall, IGF2BP3 influences the expression of malignancy-associated RNAs by modulating their interactions with miRNAs through multiple and complex mechanisms, including the recently identified effect on miRNA maturation (Wang et al., 2019). During this process, IGF2BP3 competes with the ribonuclease Drosha to bind to pri-miRNAs in the nucleus, thus avoiding miRNA maturation and indirectly favoring the stability of miRNA transcript targets.

Beyond the interaction with miRNAs, evidence from the literature indicates that the mechanism of action of IGF2BP3 also relies on its direct interaction with protein partners. Studies of immunoprecipitation followed by mass spectrometry demonstrated specific functional interactions between IGF2BP3 and (1) enzymes (helicases, deubiquitinases or ribonucleases); (2) nuclear ribonucleoproteins; and (3) stress granule-associated proteins. Particularly, IGF2BP3 can directly interact with the ribonuclease XRN2 (Mizutani et al., 2016), or the deubiquitinase ubiquitin-specific peptidase 10 (USP10) (Zhao et al., 2017), causing *EIF4EBP* mRNA or p53 protein degradation, respectively. How IGF2BP3 recruits its protein partners onto transcript targets still needs to be elucidated. On the other hand, interaction with the nuclear ribonucleoprotein (nRNP) HNRNPM was found to be crucial for the specific localization of IGF2BP3 within the nucleus and, indirectly, for the stability of IGF2BP3 transcript targets (Rivera Vargas et al., 2014). Much can still be uncovered regarding the functional effects of the interaction between IGF2BP3 and its protein partners. Indeed, multiple partners interacting with IGF2BP3 have been identified, including stress granule-associated proteins (G3BP1 and G3BP2) (Zhao et al., 2017). IGF2BP3 acts within RNP granules in a dynamic process that likely occurs through the polymerization of low-complexity sequences present in RBPs (Kato and Nakamura, 2012; Hentze et al., 2018), leading to the aggregation and recruitment of hundreds of RBP molecules and 10–30 mRNA transcripts into these granules (Jonson et al., 2007; Huang et al., 2018a). Stress granules represent cytoplasmic protein/RNA aggregates in which mRNAs are stored during stress conditions, such as nutrient deprivation, hypoxia, and oxidative stress. IGF2BP1 and IGF2BP2 have been previously reported to participate in stress granule formation in mammalian cells under oxidative stress conditions (Tourriere et al., 2003; Kedersha et al., 2016; for a review, see Protter and Parker, 2016). Similarly, IGF2BP3 may interact with G3BPs or TIAR stress granule-associated proteins, determining the formation of stress granules, under specific conditions, including stress exposure or, interestingly, mRNA transport (Taniuchi et al., 2014a, b; Huang et al., 2018b). In HeLa cells exposed to heat shock, the mRNA stability of the IGF2BP3 target *MYC* was significantly higher in cells with forced IGF2BP3 expression than that in control cells, demonstrating the protective effect of IGF2BP3 on its targets during stress (Huang et al., 2018b). In contrast, in pancreatic cancer cells, IGF2BP3 is assembled in stress granules for the transportation of its target RNAs along microtubules toward cell protrusions, thus favoring the local translation of cell migration-related transcripts (Taniuchi et al., 2014a, b). Therefore, IGF2BP3-mediated mRNA storage within stress granules represents a further mechanism underlying the enhancement of mRNA stability and the safe transport of RNAs within subcellular compartments.

Notably, while most of the studies reported in the literature investigate the regulation of coding RNA, IGF2BP3 also interacts with non-coding RNAs, including miRNAs (Samanta et al., 2018), lncRNAs (Li et al., 2018) and the new class of circular RNA (circRNA; Box 1; Schneider et al., 2016). In particular, IGF2BP3 can exert its effects by destabilizing miR145-5p, thus favoring the function of breast cancer stem cells (CSCs), or by stabilizing the lncRNA LINC01138, thus sustaining the proliferation and invasion abilities of hepatocellular carcinoma cells. Interestingly, at least 34 IGF2BP3-associated circRNAs, including the circRNAs CDYL, NFATC3, and ANKRD17, have been recently identified; however, the functional effects of these interactions are still unknown.

## Effects of IGF2BP3 on Tumor Progression

IGF2BP3 has been implicated in various aspects of human tumor progression regulating cell growth, migration, and the response to drugs. These effects largely depend on the cellular context and presence of target transcripts. Some examples are provided in relation to the different cellular processes, but it is necessary to acknowledge that functional connections strictly require dedicated studies. This section of the review highlights the multiplicity of the mechanisms and targets that have been described in different tumors thus far, but mounting evidence indicates a complex scenario that may change dynamically as tumor cells become more aggressive and/or interact with tumor microenvironment components. To render this sweeping information, which includes different effects in different cellular contexts, more clear for the readers, a summary of to date reported IGF2BP3 targets in cancer is reported in Box 2.

**BOX 2. List of IGF2BP3 targets.**

**Table:** 

ABCF1	CD164	LINC01138
ABCG2	c-myc	LIN28
ARF6	COX2	MMP9
ARHGEF4	EIF4EBP2	PDPN
CCND1	HMGA2	Slug
CCND3	hsa-miR-145-5p	TP53
CCNG1	hsa-miR-3614	ULBP2
CDK6	IGF1R	
CD44	IGF2	

**At the experimental level**, IGF2BP3 sustains cancer cell growth and proliferation while putatively inhibiting apoptosis. As stated above, a well-established mechanism of action of IGF2BP3 is based on its protection from Let-7 miRNA-mediated decay. In this landscape, IGF2BP3 sustains the expression of *HMGA2* (Jonson et al., 2014), a DNA-binding protein that cooperates with the transcription machinery to alter the chromatin structure (De Martino et al., 2019), thus enhancing fibrosarcoma cells proliferation *in vitro*. In addition to HMGA2, Let-7 miRNAs directly repress a pantheon of well-known oncogenes, such as RAS, MYC, LIN28, and IGF1R, and cell cycle factors, such as cyclin D1 and cyclin D2 (Chirshev et al., 2019), most of which are indeed described as targets of IGF2BP3 (Jonson et al., 2014; Rivera Vargas et al., 2014; Palanichamy et al., 2016; Mancarella et al., 2018a) with a key role in cell proliferation. It can be speculated that through protection against Let-7 miRNA, IGF2BP3 favors the stability and translation of (1) *IGF1R* mRNA, thereby affecting the constitutive activation of its intracellular pathway and the *in vitro* growth of hepatocellular carcinoma or Ewing sarcoma cells (Fawzy et al., 2016; Mancarella et al., 2018a); (2) *MYC* and *CDK6* transcripts, thereby promoting the proliferation of hematopoietic stem and progenitor cells in mice (Palanichamy et al., 2016). In addition, by upregulating LIN28, which also enhances the expression of IGF2, histone H2a, cyclin A, cyclin B, and CDK4 (Balzeau et al., 2017), IGF2BP3 can establish complex, positive-feedback loops that further facilitate tumor cell growth and malignancy. Overall, a direct interaction between IGF2BP3 and IGF2, with a consequent promotion of cell proliferation *in vitro*, has been demonstrated in leukemia (Liao et al., 2005), thyroid cancer (Panebianco et al., 2017), and glioma (Suvasini et al., 2011). In contrast to evidence in normal embryonic tissues, IGF2BP3 promotes *IGF2* mRNA translation in cancer cells by binding its 3′-UTR, leading to the increased activation of IGF signaling. In addition, IGF2BP3 contributes to stabilizing *COX-2* mRNA, favoring the translation of this crucial mediator of inflammation and antiapoptotic signals in leukemia cells (Ko et al., 2016). However, in the latter cases, the exact mechanism of action elicited by IGF2BP3 on these targets has not been investigated. It can only be speculated that binding the 3′-UTRs, IGF2BP3 protects these targets from miRNA-mediated decay.

IGF2BP3 has also been found to repress RNAs and miRNAs. This effect has been described in lung and cervical cancer cells in the case of *EIF4E-BP2*, which encodes a negative regulator of eukaryotic translation initiation factor 4E (eIF4E) (Mizutani et al., 2016), and miR145-5p in breast cancer (Samanta et al., 2018). The repression of EIF4E-BP2 through the IGF2BP3-mediated recruitment of ribonucleases within RNPs promotes the proliferation of cancer cells (Mizutani et al., 2016); the IGF2BP3-induced destabilization of miR145-5p favors the expression of WNT5B, which activates TAZ, a transcriptional coactivator of Hippo signaling necessary for the function of breast cancer CSCs (Samanta et al., 2018). A putative role of IGF2BP3 in self-renewal and tumor initiation, i.e., two properties associated with CSCs, has been suggested in several tumors through different mechanisms. As mentioned, IGF2BP3 regulates the expression of HMGA2 and LIN28, allowing the symmetrical division of CSCs thus sustaining their stemness-like phenotypes (Jonson et al., 2014; Puca et al., 2014; Balzeau et al., 2017). IGF2BP3 recruits the deubiquitinase USP10 thereby attenuating p53 protein stability and increasing tumorigenicity of lung cancer cells *in vivo* (Zhao et al., 2017). In addition, IGF2BP3 sustains *SNAI* (*Slug*) mRNA translation in breast cancer cells (Samanta et al., 2016), putatively preventing its miRNA-mediated decay; in turn, SNAI favors the transcription of the stem cell factor SOX2. Consistent with a putative role in the maintenance of cellular stemness, IGF2BP3 expression is higher in triple-negative breast CSCs (Samanta et al., 2016) and hepatocellular carcinoma tumor-initiating stem-like cells (Chen et al., 2013) than in the entire population of tumor cells. In hepatocellular carcinoma, the IGF2BP3/AKT/mTOR pathway inactivates TGF-β signaling to maintain the expression of pluripotency genes along with the tumorigenesis and chemoresistance of CD133(+) stem cells (Chen et al., 2013). Altogether, these observations highlight a putative role of IGF2BP3 in promoting or preserving tumor cell subpopulations with stem cell features, thereby contributing to tumor establishment and progression.

In addition, IGF2BP3 promotes cell migration. Compared to the phenotype of IGF2BP3-null cells, IGF2BP3-expressing tumor cells display a marked motility-prone phenotype with an adherent shape, cellular extensions, lamellipodia, frequent cell–cell adhesion contacts (Vikesaa et al., 2006) and an increased capability to form metastases *in vivo* (Zhao et al., 2017; Mancarella et al., 2018b). Accordingly, different mediators of cell migration/invasion and cell adhesion have been reported as IGF2BP3 mRNA targets in different tumor types. Besides favoring cell proliferation, IGF1R and HMGA2 also regulate cell migration (Sheen et al., 2015; Mancarella et al., 2018a). Other described mediators of IGF2BP3-induced cellular motility include the type IV collagenase MMP9, which drives the degradation of the basement membrane and promotes the release of growth factors from the extracellular matrix, and the cell surface receptor of sialomucin, i.e., CD164 (endolyn), which is involved in cell adhesion (Hafner et al., 2010; Samanta et al., 2012). These mediators were identified as IGF2BP3 targets by PAR-CLIP studies and were subsequently validated in triple-negative breast cancer cells; however, the exact mechanisms of IGF2BP3-mediated regulation are still unknown. Furthermore, IGF2BP3 has been shown to bind to the 3′-UTR and sustain the expression of the hyaluronan receptor CD44 and the epithelial adhesion protein podoplanin (PDPN), which interact with actin and promote invadopodia formation (Vikesaa et al., 2006; Hwang et al., 2012).

However, large-scale genomic approaches identified an IGF2BP3-RNA interaction network of 164 transcripts associated with cellular migration, cell adhesion, actin cytoskeleton remodeling, and invadopodia formation; these transcripts include mRNAs previously identified by other authors, thereby indicating the existence of a complex scenario (Ennajdaoui et al., 2016).

Local translation of RNAs is required for cell migration (Mofatteh and Bullock, 2017). In pancreatic cancer cells, IGF2BP3 and IGF2BP3-bound transcripts, including *ARF6* and *ARGHEF4*, accumulate in membrane protrusions (Taniuchi et al., 2014a). This accumulation is due to the activity of the motor kinesin protein KIF20A (Taniuchi et al., 2014b), which transports IGF2BP3 and its target transcripts toward cell protrusions along microtubules, leading to the local translation of mRNA into proteins that favor the formation of membrane protrusions and cell motility (Taniuchi et al., 2014a).

Other experimental evidence indicates that IGF2BP3 regulates the response to anticancer treatments. By regulating Lin28, HMGA2, CD44, IGF2, and IGF1R, IGF2BP3 increases cell survival and resistance to conventional and targeted drugs in several tumors. In particular, by affecting IGF2 and/or IGF1R expression, IGF2BP3 has been shown to modulate sensitivity to anti-IGF1R agents (Liu et al., 2017; Panebianco et al., 2017; Mancarella et al., 2018a) and MAPK/PI3K inhibitors (Suvasini et al., 2011). Moreover, IGF signaling modulation is thought to be responsible for the association between IGF2BP3 expression and radio-resistance in chronic myeloid leukemia and squamous cell esophageal cancer (Liao et al., 2011; Yoshino et al., 2014). In ovarian cancer, the overexpression of IGF2BP3 and LIN28 has been associated with cisplatin resistance, which was attributed to the downregulation of hCTR1 (a transmembrane protein that imports cisplatin into mammalian cells), and was found to be responsible for a poor outcome (Hsu et al., 2015). In triple-negative breast cancer cells, an IGF2BP3 depletion increased cell sensitivity to doxorubicin and mitoxantrone (Samanta et al., 2013). This effect was due to the IGF2BP3-mediated stabilization of *ABCG2* mRNA, which is an ATP-binding cassette (ABC) transporter and a major effector of drug resistance (for a review, see Robey et al., 2018).

Limited but interesting evidence indicates that IGF2BP3 affects the interaction with the tumor microenvironment. Cancer cells must face harsh microenvironmental conditions, including hypoxia, nutrient-deprivation, space constraints, oxidative stress and the immune response, to remain viable. In the immune response, interactions between tumor and immune cells represent a major determinant of cancer behavior. Schmiedel et al. (2016) demonstrated that IGF2BP3 favors the immune escape of cancer cells by inhibiting the cytotoxic effect mediated by natural killer cells through the promotion of mRNA decay of the stress-induced ligand ULBP2. In the interaction between tumor cells and the tumor microenvironment, secreted molecules represent crucial mediators of local and systemic cellular communication. Interestingly, evidence insinuating that tumor cells are able to release IGF2BP3 in the extracellular compartment is based on recent findings demonstrating the presence of IGF2BP3 in the serum of cancer patients (Szarvas et al., 2014; Tschirdewahn et al., 2019). However, the molecular mechanisms underlying this evidence are still unknown. In particular, it is still not clear how IGF2BP3 is released and whether circulating IGF2BP3 can still elicit functional malignant effects.

**At the clinical level**, as also reviewed by Lederer et al. (2014), IGF2BP3 is expressed *de novo* in a variety of tumor types unlike normal tissues (Figure 2). Table 1 summarizes those tumor types that, to the best of our knowledge, display an higher expression of IGF2BP3 compared to normal counterpart and where IGF2BP3 has been suggested as a diagnostic and/or prognostic biomarker. Of those, the IGF2BP3-positive tumors generally display high metastatic behavior and poor outcome as well as increased tumor size, advanced tumor stage and lymph node metastasis.

**TABLE 1:** IGF2BP3 participation in human tumors.

**Cancer types**	**Diagnosis**	**Prognosis**	**References**
**Solid tumors**			
Skin			
Squamous cell carcinoma	✓		Richey et al. (2018)
Melanoma		✓	Sheen et al. (2016)
Lung			
Lung adenocarcinoma		✓	Yan et al. (2016)
Non-small cell lung cancer		✓	Shi et al. (2017)
Neuroendocrine tumor of lung		✓	Del Gobbo et al. (2014)
Malignant peritoneal mesothelioma		✓	Hui et al. (2018)
Breast			
Triple-negative breast carcinoma		✓	Ohashi et al. (2017)
Pancreatic and Gastrointestinal tract			
Intraductal papillary mucinous neoplasm of pancreas	✓		Senoo et al. (2018)
Pancreatic ductal adenocarcinoma	✓	✓	Johnson et al. (2016); Aksoy-Altinboga et al. (2018)
Gastroenteropancreatic neuroendocrine neoplasia		✓	Er et al. (2017)
Colorectal cancer	✓	✓	Wei et al. (2017); Xu et al. (2019)
Esophageal adenocarcinoma		✓	Plum et al. (2018)
Gastric cancer		✓	Lee et al. (2017)
Prostate			
Prostate cancer		✓	Szarvas et al. (2014)
Cervix and uterus			
Ovarian serous carcinoma		✓	Mohanty et al. (2019)
Adenocarcinoma *in situ* of the uterine cervix	✓		Li et al. (2007a)
Endometrial serous carcinoma	✓		Li et al. (2007b)
Bladder and kidney			
Urothelial carcinoma		✓	Yang et al. (2019)
Renal cell carcinoma		✓	Tschirdewahn et al. (2019)
Liver			
Hepatocellular carcinoma		✓	Hu et al. (2014)
Intrahepatic cholangiocarcinoma		✓	Gao et al. (2014)
Head and neck			
Oral squamous cell carcinoma		✓	Tarsitano et al. (2016)
Bone and soft tissues			
Ewing sarcoma		✓	Mancarella et al. (2018b)
Leiomyosarcoma	✓	✓	Cornejo et al. (2012); Yasutake et al. (2018)
Brain			
Astrocytoma		✓	Barton et al. (2013)
Glioma		✓	Del Gobbo et al. (2015)
Neuroblastoma		✓	Chen et al. (2011)
**Hematological tumors**			
Leukemia			
B-acute lymphoblastic leukemia	✓		Palanichamy et al. (2016)
Myeloma			
Multiple myeloma			Canella et al. (2015)
Lymphoma			
Mantle cell lymphoma			Hartmann et al. (2012)
Hodgkin lymphoma	✓		Tang et al. (2013)

This is not surprising since, as stated above, IGF2BP3 increases cell proliferation while blocking apoptosis and favoring stemness, migration and drug resistance. A direct correlation has been reported between the immunohistochemical evaluation of IGF2BP3 expression and increased staining of the proliferation index ki67 in malignant peritoneal mesothelioma (Hui et al., 2018), neuroendocrine tumors of the lung, in which IGF2BP3 was also directly correlated with the stem cell marker Nanog (Del Gobbo et al., 2014), and triple-negative breast carcinoma, in which IGF2BP3 expression was also significantly associated with a poor response to neoadjuvant chemotherapy (Walter et al., 2009; Ohashi et al., 2017). The combined evaluation of IGF2BP3 and the proapoptotic protein BCL2 was found to be particularly effective for diagnosis in squamous cell carcinoma (Richey et al., 2018). In hepatocellular carcinoma, the copresence of IGF2BP3 and its target CD44 is correlated with advanced tumor stage/grade and metastasis (Wachter et al., 2012; Hu et al., 2014), while in Ewing sarcoma patients, contemporary high expression of IGF2BP3 and low expression of its counteracting partner ABCF1 is correlated with a particularly poor outcome (Mancarella et al., 2018b). In addition, the circulating IGF2BP3 protein levels, recently detected in serum from prostate cancer and renal cell carcinoma patients, were found to be associated with a significantly higher risk of cancer-specific death or relapse (Szarvas et al., 2014; Tschirdewahn et al., 2019). These findings demonstrate that the IGF2BP3-induced phenotypic effects observed *in vitro* can be recapitulated in clinical specimens and that it is possible to exploit this knowledge in the clinical settings for monitoring tumor progression.

## Potential Relevance of IGF2BP3 in Therapy

Based on its absence in normal tissues, with very few exceptions, IGF2BP3 represents a putative valuable and specific target for cancer therapy.

To date, no direct inhibitor of IGF2BP3 activity has been developed. However, the “druggability” of RBPs has been recently demonstrated for Musashi1 or HuR. In particular, the small molecule luteolin was observed to interfere with the RNA-binding capacities of Musashi1 by blocking its RNA-binding pocket (Yi et al., 2018). Similarly, multiple compounds, including the recently described dihydrotanshinone-I that directly blocks the RNA-binding domains of HuR, prevent its association with target RNAs (Lal et al., 2017). In these two cases, the blockades of RBP activity correlated with reduced *in vitro* proliferation, viability, and migration or decreased xenograft tumor growth, respectively, demonstrating the putative effectiveness of these approaches. Nevertheless, a deeper biochemical comprehension of the IGF2BP3 RNA-binding properties is needed for the successful development of direct inhibitors of its functions.

In contrast, consistent preclinical studies have provided pharmacological options to block IGF2BP3 expression. The use of an isocorydine derivative (d-ICD), i.e., an alkaloid monomer purified from *Papaveraceae* sp. plants, has been demonstrated to inhibit IGF2BP3 expression and reduce the growth of hepatocellular carcinoma cells (Li et al., 2015). In addition, inhibitors of bromodomain and extraterminal domain (BET) proteins, such as JQ1 or iBET, have been found to downregulate IGF2BP3 expression and its targets in Ewing sarcoma and B cell acute lymphoblastic leukemia, consequently attenuating tumor growth (Palanichamy et al., 2016; Elagib et al., 2017; Mancarella et al., 2018b). Considering that clinical trials using BET inhibitors have been performed in hematological and solid tumors with an observed manageable toxicity (Amorim et al., 2016; Berthon et al., 2016), these agents may represent a concrete treatment option for patients with high levels of IGF2BP3 (Mancarella and Scotlandi, 2018; Mancarella et al., 2018b).

IGF2BP3 has also been postulated as a potential vaccine candidate. Studies investigating lung cancer have shown that IGF2BP3 is immunogenic as assessed by the presence of an antibody against recombinant IGF2BP3 in lung pleural effusions (Wang et al., 2003), and immunogenic peptides derived from IGF2BP3 induce tumor-reactive and human leukocyte antigen (HLA)-A2 (A^∗^02:01)-restricted cytotoxic T lymphocytes (CTL) (Tomita et al., 2011). More recently, cancer vaccination using the IGF2BP3 508-516 peptide along with the LY6K 177-186 and CDCA1 56-64 peptides was tested in a phase II open-label, non-randomized clinical trial in head and neck squamous cell cancer patients, indicating that a vaccination-induced immune response was positively correlated with a better prognosis (Yoshitake et al., 2015).

Considering the recent evidence concerning the epigenetic regulation of IGF2BP3 or posttranslational modification mediated by the PI3K/Akt or MAPK pathways, it is plausible that agents capable to influence the activity of epigenetic factors, such as inhibitors of DNA methyltransferases or histone deacetylase inhibitors, and targeted therapies that block specific intracellular signaling pathways may affect IGF2BP3 activity. However, more detailed preclinical studies are required before these drugs can be considered a concrete possibility.

## Critical Issues and Perspectives

Although the molecule has been largely described to impact tumor initiation and progression, there is lack of knowledge regarding relevant issues. In particular, the following issues require further investigation:

1. The physiological role of IGF2BP3 still needs to be clearly elucidated.
2. Although there is clear support showing that IGF2BP3 plays a direct role in tumorigenesis and cancer progression, the mechanisms by which IGF2BP3 elicits its effects are incompletely understood. Importantly, the molecular mechanisms underlying the IGF2BP3-mediated regulation of non-coding RNAs still need elucidation.
3. The impact of IGF2BP3 on tumor predisposition is still obscure.
4. The precise discrimination of the specific properties of IGF2BP3 in relation to the other IGF2BP members is difficult due to the high level of homology but is necessary to fully exploit the clinical potential of these molecules.
5. The identification of high-quality and highly paralog-specific antibodies is mandatory for their proper use in the clinic as tissue and/or circulating biomarkers. In addition, adequate tools to study the intracellular modifications of IGF2BP3 are required.
6. The presence of IGF2BP3 in the plasma offers novel interesting clues. More studies are required to test the clinical value of IGF2BP3 as a circulating biomarker of risk and response.
7. A deeper understanding of the posttranslational modification and phosphorylation of IGF2BP3 is highly desirable as it may open new avenues for therapy.
8. The interaction between IGF2BP3 and the tumor microenvironment is still poorly described.
9. The role of IGF2BP3 in cell metabolism is still unknown. Although IGF2, IGF1R, and LIN28 have been described as target of this RBP, the overall impact of IGF2BP3 on glycose metabolism and insulin-induced signaling has not been assessed.

## Concluding Remarks

IGF2BP3 represents an intriguing posttranscriptional factor in tumor malignancy. Important advancements have been achieved over the last years concerning our understanding of the oncogenic processes driven by RBPs, revealing that the relevance of these regulators in tumorigenesis and cancer progression has been largely underscored. Regarding IGF2BP3, the information obtained to date indicates a complex scenario in which this molecule acts through multiple and highly cell type-dependent contexts. The molecule is able to influence the expression of all RNA species, thus driving key malignant processes in cancer cells. In addition, interactions between IGF2BP3 and the tumor-microenvironment have been identified, highlighting a novel putative function in the interplay between tumor and normal cells. Experimental and clinical findings indicate that the evaluation of IGF2BP3 expression and its targets may concur to address the clinical need of new biomarkers for the risk-based stratification of patients at diagnosis and may offer innovative treatment opportunities. However, the clinical use of this molecule is still far from being a concrete possibility due to the many molecular and technical issues that remain unsolved.

## Author Contributions

All authors listed have made a substantial, direct and intellectual contribution to the work, and approved it for publication.

## Conflict of Interest

The authors declare that the research was conducted in the absence of any commercial or financial relationships that could be construed as a potential conflict of interest.
